# Intra-Articular Application of Autologous, Fat-Derived Orthobiologics in the Treatment of Knee Osteoarthritis: A Systematic Review

**DOI:** 10.3390/cells13090750

**Published:** 2024-04-25

**Authors:** Matthias Holzbauer, Eleni Priglinger, Stig-Frederik Trojahn Kølle, Lukas Prantl, Christian Stadler, Philipp Wilhelm Winkler, Tobias Gotterbarm, Dominik Duscher

**Affiliations:** 1 Department for Orthopedics and Trauma Surgery, Med Campus III, Kepler University Hospital, Krankenhausstrasse 9, 4020 Linz, Austria; eleni.priglinger@jku.at (E.P.); christian.stadler@kepleruniklinikum.at (C.S.); philipp.winkler@kepleruniklinikum.at (P.W.W.); tobias.gotterbarm@kepleruniklinikum.at (T.G.); 2 Faculty of Medicine, Johannes Kepler University Linz, Altenbergerstraße 69, 4040 Linz, Austria; 3 Cerix, StemMedical, Strandvejen 191, 2900 Copenhagen, Denmark; sfk@stemform.com; 4 Department of Plastic, Hand and Reconstructive Surgery, University Hospital Regensburg, Franz-Josef-Strauss-Allee 11, 93053 Regensburg, Germany; lukas.prantl@klinik.uni-regensburg.de (L.P.); dominikduscher@me.com (D.D.); 5 TF Plastic Surgery and Longevity Center, Herzogstrasse 67, 80803 Munich, Germany and Dorotheergasse 12, 1010 Vienna, Austria

**Keywords:** adipose tissue-derived stromal cells, cartilage regeneration, knee osteoarthritis, mesenchymal stem cells, orthobiologics, stromal vascular fraction

## Abstract

The aim of this study was to review the current literature regarding the effects of intra-articularly applied, fat-derived orthobiologics (FDO) in the treatment of primary knee osteoarthritis over a mid-term follow-up period. A systematic literature search was conducted on the online databases of Scopus, PubMed, Ovid MEDLINE, and Cochrane Library. Studies investigating intra-articularly applied FDO with a minimum number of 10 knee osteoarthritis patients, a follow-up period of at least 2 years, and at least 1 reported functional parameter (pain level or Patient-Reported Outcome Measures) were included. Exclusion criteria encompassed focal chondral defects and techniques including additional arthroscopic bone marrow stimulation. In 28 of 29 studies, FDO showed a subjective improvement in symptoms (pain and Patient-Reported Outcome Measures) up to a maximum follow-up of 7.2 years. Radiographic cartilage regeneration up to 3 years postoperatively, as well as macroscopic cartilage regeneration investigated via second-look arthroscopy, may corroborate the favorable clinical findings in patients with knee osteoarthritis. The methodological heterogeneity in FDO treatments leads to variations in cell composition and represents a limitation in the current state of knowledge. However, this systematic review suggests that FDO injection leads to beneficial mid-term results including symptom reduction and preservation of the affected joint in knee osteoarthritis patients.

## 1. Introduction

Total joint arthroplasty is an established and beneficial therapy for advanced stages of knee osteoarthritis (OA) with satisfaction rates of around 80%. However, revision rates of up to 12% after 10 years of follow-up have been reported causing substantial loss of function. Additionally, it was already shown by event simulation, that 21% of all total knee replacements could be avoided, by state-of-the-art cartilage repair procedures [1]. Therefore, researchers worldwide are striving to promote joint regeneration and cartilage healing. Attempts to study cartilage healing date back to Pridie, who detected “a method of resurfacing osteoarthritic knee joints” via drilling knee cartilage lesions in 1959 [2] and Steadman et al. who refined this concept of facilitating the differentiation of bone marrow mesenchymal stem cells (MSCs) to functional fibrocartilage using arthroscopic microfracture [3].

In 1999, Pittenger et al. managed to isolate MSCs from bone marrow for the first time, proposing their potential to differentiate into adipocytes, osteoblasts, and chondrocytes [4]. MSCs have since been extensively studied and can be isolated from various sources, including the umbilical cord, adipose tissue, synovial membrane, placenta, cartilage, and skeletal muscle [5]. In 2001, Zuk et al. were the first to characterize adipose-derived MSCs from autologous subcutaneous fat tissue which are commonly referred to as adipose-derived stromal/stem cells (ASC) [6]. In 2011, Pak et al. published the first-in-human case series of knee and hip OA patients treated with ASC following encouraging animal experiments [7]. The clinical use of adipose-tissue-based injections for cartilage regeneration can be summarized by the term “fat-derived orthobiologics” (FDO). However, the procession of lipoaspirate after liposuction for the purpose of intra-articular injection varies widely in the current literature. Due to terminological inhomogeneity in the medical literature, the following terminology was proposed: Enzymatic separation of lipoaspirate, which is widely performed as described by Zuc et. al., was called cellular SVF (cSVF) [8,9]. If the cell population of cSVF is further incubated in a culture medium for in vitro expansion of ASC, the subsequent injection is called ASC therapy [10]. To distinguish the cell populations of cSVF and ASC, they must contain a minimum of 70% and 90% viable cells, respectively, as well as a minimum frequency of 1% or 5% of fibroblastoid colony-forming units, respectively [11]. By contrast, mechanically processed lipoaspirate is called tissue SVF (tSVF) [8,9].

In the past decade, several favorable outcomes resulting from intra-articularly applied, autologous FDO have been reported and summarized in systematic reviews [9,12,13,14]. While the majority of reviews concentrate on certain subcharacteritics of FDO, the literature lacks an investigation into the sustainability or functional outcome of an FDO treatment after a mid-term follow-up period.

To fill this literature gap, this systematic review aims to screen the current literature for studies with a minimum of 2 years of follow-up investigating intra-articularly applied, autologous FDO as a standalone procedure on functional parameters in treating primary knee OA. Studies combining FDO with arthroscopic bone marrow stimulation were excluded from this review.

## 2. Materials and Methods

This review was conducted according to the Preferred Reporting Items for Systematic Reviews and Meta-Analyses guidelines and registered in the Inplay Register [15].

### 2.1. Eligibility Criteria

To assess the current state of knowledge on intra-articular injection of FDO, studies on patients suffering from primary knee OA were identified. Because FDO is a regenerative approach to a progressive disease, a minimal follow-up period of 2 years was deemed reasonable to monitor sustainable treatment outcomes.

The inclusion criteria for this review were as follows:

- Patients with primary knee OA;
- Autologous, processed or non-processed, intra-articular fat tissue injection including cSVF, tSVF, and ASC;
- Mean or median follow-up period of more than 2 years with minimum one clinical parameter (visual analogue scale (VAS) for pain or Patient-Reported Outcome Measures (PROMs)) available;
- Minimal number of 10 patients.

The following exclusion criteria were defined:

- Patients with focal chondral defects;
- Additional arthroscopic bone marrow stimulation (microfracture or drilling).

Randomized controlled trials (RCTs), prospective or retrospective cohort studies, or case series as study types were included. In contrast, conference abstracts, clinical trial entries, editorials, and commentaries were excluded, because they lack of required details or parameters assessed in this review.

### 2.2. Literature Search Strategy

To identify relevant studies, a systematic literature search was implemented until the 1st of November 2023. The search was conducted on the online databases of Scopus, PubMed, Ovid MEDLINE, and Cochrane Library. All studies in English and German language were included. The search algorithm (in the title and abstract) is presented in Table 1. The term “knee” was not incorporated in the search algorithm to detect studies encompassing various types of knee OA (uni-, bi- or tricompartimental).

### 2.3. Identification and Selection of Eligible Studies

First, duplicates were manually consolidated. The first author screened the titles and abstracts of all references. Studies not meeting the inclusion criteria were excluded. Following this, full texts of the included references were obtained. The first and last authors screened the full texts and re-assessed the above-mentioned inclusion and exclusion criteria.

### 2.4. Data Extraction Process and Data Items

The first and last authors extracted the following parameters from the full texts: study design, study population, follow-up period, and details regarding the technique including procession of the fat tissue (mechanically or enzymatically with or without culture; days between harvest and implantation were given in brackets in case they were not performed during the same procedure) were noted. Moreover, the injection of additional substances was documented and articles were screened whether the FDO was administered intra-articularly or implanted focally into the arthritically degenerated cartilage. If available, visual analogue scale (VAS) values for pain and PROMs were noted. If these parameters were presented using diagrams, the approximate median or mean value was given using a “≈” symbol. If the included studies report a radiological follow-up examination, the imaging modality (MRI or X-ray) used and the number of patients at the longest follow-up appointment were recorded. Furthermore, the articles were screened for a second-look arthroscopy.

All included studies pertaining to FDO injection were extracted in duplicate by the two reviewers, while a third reviewer resolved any discrepancies. The data were descriptively presented using a table.

### 2.5. Quality Assessment

The methodology of all included studies was assessed according to the recommendations of the Oxford Centre for Evidence-Based Medicine concerning the level of evidence (LoE) [16]. The methodological quality (MQ) of the studies was evaluated based on the respective study type. RCTs were rated using the modified Jadad scale, which ranges from 0 to 8 points [17]. Non-randomized studies were assessed using the Methodological Index for Nonrandomized Studies (MINORS) score [18]. For non-comparative studies, this score ranges from 0 and 16 points. In the case of comparative studies, this score incorporates an additional domain, resulting in a range between 0 and 24 points. The maximum score indicates the ideal assessment for each assessment. Both the first and the last author assessed the level of evidence and the quality of the studies, while any discrepancies were also resolved by a third reviewer.

### 2.6. Data Synthesis

Due to the methodological inhomogeneity of various FDO techniques, the above-mentioned parameters were summarized and tabulated primarily in a descriptive manner. Even a comparison of outcome parameters between the categories cSVF, ASC, and tSVF is associated with a high risk of bias. Thus, the number of patients treated with cSVF, ASC, or tSVT was calculated. Moreover, a geographic analysis was conducted aiming to indicate how many patients were included in FDO studies on every continent.

## 3. Results

**Figure 1:**
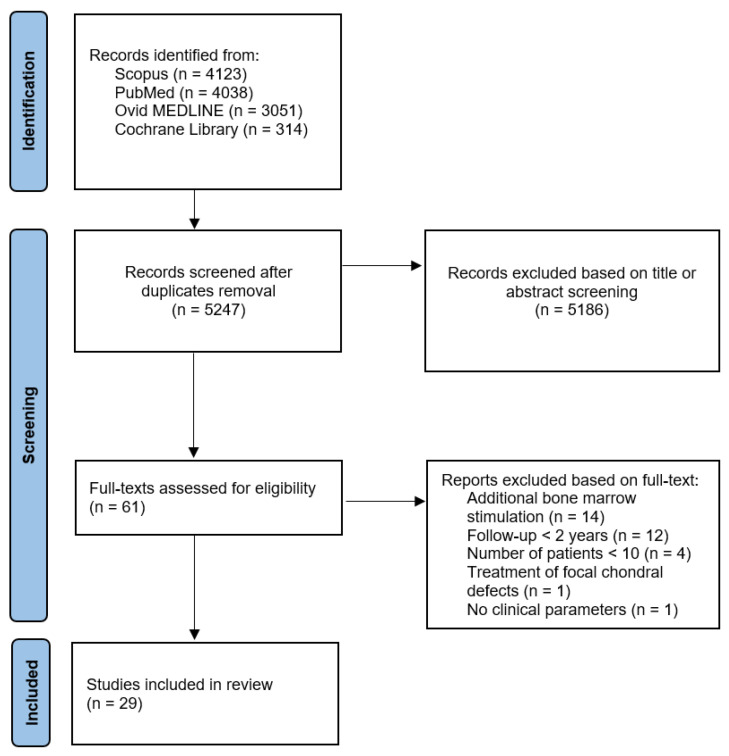
PRISMA flowchart indicating the review process.

In total, 4123 records in Scopus, 4038 records in PubMed, 3051 in Ovid MEDLINE and 314 records in the Cochrane Library were identified, comprising a total of 5247 articles screened in this review after duplicates were removed. Ultimately, the study selection process resulted in 29 papers assessed in this review (see Figure 1).

**Figure 2:**
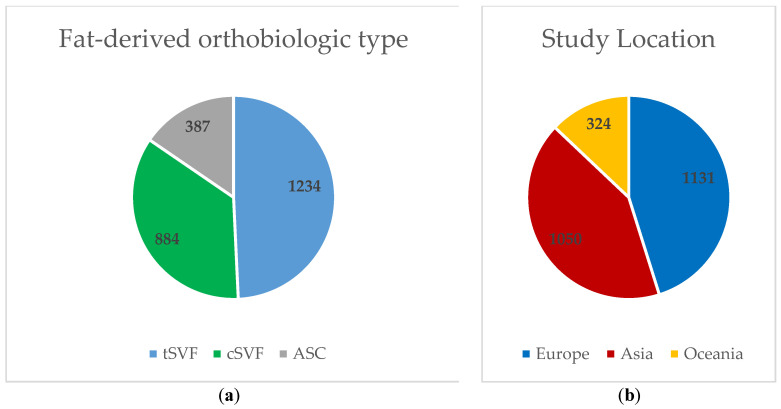
Patients categorized by (**a**) fat-derived orthobiologic type and (**b**) continent. Legends: ASC (adipose tissue-derived stromal/stem cells); cSVF (cellular stromal vascular fraction); tSVF (tissue stromal vascular fraction).

Figure 2 depicts the overall number of patients included in this review by FDO type and continent of the study’s location. All studies from the continent of Oceania included patients with ASC therapy. With the exception of one study comparing tSVF to ASC [19], all studies conducted in Asia used either ASC or cSVF.

**Figure 3:**
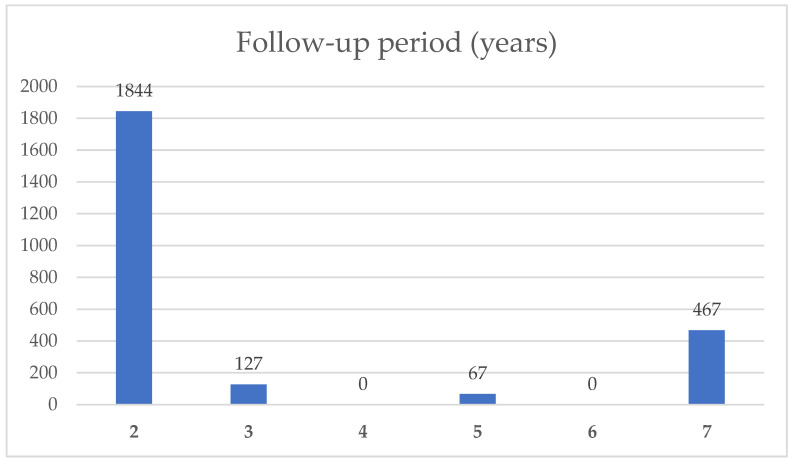
Number of patients included in this review with their follow-up period.

Of the 29 included publications, 28 investigated medial and/or lateral knee OA, while 1 study investigated patellofemoral OA [20]. In Figure 3, the cumulative number of patients with their respective follow-up period is indicated. Brief summaries of these studies were given in Table 2, while being sorted according to their mean follow-up periods. The included studies investigate patients with a wide range of OA severity, ranging from a Kellgren–Lawrence grade 0 to IV [21]. The abdomen (*n* = 19), followed by the gluteal region (*n* = 11), thigh region (*n* = 2), the flank (*n* = 2), and the infrapatellar fat pad (*n* = 1) were the chosen harvest sites of adipose tissue. Three studies did not contain the harvest site [19,22]. Three studies were presented using two rows in Table 2 because they compared two different FDO cohorts or methods: Kim et al. in two articles compared the application with and without the use of fibrin glue as a scaffold for arthroscopically guided application [23,24], and Yokota et al. compared the outcomes of tSVF and ASC [19]. In total, Table 2 contains 14 tSVF, 13 cSVF, and 5 ASC cohorts. Kim et al. used fibrin glue as a scaffold in five studies [23,24,25,26,27], while platelet-rich plasma (PRP) was injected as an additional substance in three studies [24,28,29]. Postoperative pain levels were available in 22 cohorts. Thus, pain reduction at the final follow-up examination was reported in all but one study: Screpsis et al. [30] reported a significant reduction after 6 and 12 months while pain levels were similar to baseline levels after 2 years. An improvement in functional scores could be detected in all studies. According to radiographic follow-up data, X-rays are available in 8 cohorts [19,31,32,33,34,35], while 14 studies report the effects of FDO on cartilage status via magnetic resonance imaging (MRI) [22,26,29,31,32,33,34,35,36,37,38,39]. Second-look arthroscopy was performed in four studies [23,24,28,40].

## 4. Discussion

Cell-based tissue engineering has emerged as a promising approach in the treatment of OA in the last decade. Adipose tissue has emerged as the most attractive source of MSC because of its abundance, ease of accessibility, as well as regenerative capabilities [10]. ASCs have the potential to differentiate into chondrocytes, especially when exposed to hydrostatic pressure [51], the functional characteristics of articular cartilage tissue are defined by far more than its cellular components.

When discussing the therapeutic effects of FDO, paracrine effects of ASC, i.e., the secretion of a wide range of bioactive molecules, are recently considered even more important than the differentiation into parenchymal cells [52,53]. These beneficial effects of ASC’s numerous factors and cytokines on recipient cells leading to anti-apoptosis, angiogenesis, immunomodulation, support of the differentiation and growth of local stem and progenitor cells, chemo attraction, and anti-scarring [10] are collectively known as the “secretome” theory [54,55]. In vitro studies suggest that the synovial fluid has the capacity to initialize macrophage differentiation [56]. Therefore, the paracrine effects of FDO may also have an impact on the synovial fluid niche by modulating macrophage polarization and the subsequent inflammatory response.

Based on the results of this review, FDO appears to result in subjective improvement of symptoms of knee OA. Apart from 1 study out of 29 [30], beneficial outcomes were reported throughout via pain reduction and/or improvement of functional scores. In addition to subjective scores, the effects of FDO on the articular cartilage were investigated macroscopically through arthroscopy and radiographically:

In four studies, second-look arthroscopy was used to macroscopically monitor cartilage lesions [23,24,28,40]. In three studies, the second procedure was performed 1 year postoperatively, and the chondral lesions were evaluated using the International Cartilage Repair Society Macroscopic Evaluation of Cartilage Repair (ICRS) [23,24,40]. The researchers found a significant negative correlation between ICRS repair grade and functional parameters, i.e., International Knee Documentation Committee (IKDC) subjective knee form and Tegner Activity Scale. Additionally, patients with a lower body mass index and a defect size smaller than 5.4 cm^2^ were associated with better macroscopic defect repair and better functional parameters [23,40]. Conversely, functional parameters appear to reliably indicate the macroscopic chondral status. Moreover, cellular stromal vascular fraction (cSVF) implantation using fibrin glue as a scaffold, in comparison to cSVF injection alone, showed 58–65% vs. 23–35% ICRS grade I–II chondral regeneration [23,24]. Slightly better ICRS grades could also be achieved if cSVF was injected in combination with PRP: ICRS grade I–II 35% vs. 24% [23,24,40]. In one study with second-look arthroscopy after 2 years, 62% of patients resulted in “positive” or “very positive” results, indicating at least newly forming cartilage partially covering the lesion [28].

Radiological follow-up examinations using X-ray and MRI were performed 0.5 to 5 years postoperatively. Up to 3 years of follow-up, eight studies [22,26,29,33,35,36,37,39] showed favorable signs of cartilage regeneration, while two studies [31,34] reported no changes after FDO injection. In terms of comparable MRI scores, four studies reported the MRI observation of cartilage repair tissue (MOCART) score which assesses the cartilage repair including the surrounding tissue in nine parameters with a maximal total score of 100 indicating perfect hyaline-like repair [57]: the final results were 63 after 1.5 years [58], 89 after 2 years [38], and 70 as well as 76 after 3 years [26,36]. One study showed even a progression compared to the assessment after 1 year [36]. Three studies used the Whole-Organ Magnetic Resonance Imaging Score (WORMS) ranging from 0 (completely normal joint) to 332 [59], while Zaffagnini et al. [34] showed no change after 2 years and Koh et al. proved a significant reduction after 2 years (from baseline 60 to 48 points) [25]. Kim et al. reported a reduction up to 3 years postoperatively (67 points) and recurrence to baseline values after 4 and 5 years (73 points) [32]. However, the cartilage defect area was still reduced after 5 years compared to baseline values. After 5 years, Zhang et al. [31] reported an 8% decrease in full-thickness cartilage volume, which was significantly lower compared to the hyaluronic acid (HA) group. Of all patients, 92% and 84% showed a better or constant full-thickness defect and Kellgren–Lawrence grade, respectively. Studies assessing changes in different knee compartments found the lowest regenerative potential of FDO in medial tibial cartilage defects, especially in varus knee deformities [33,35,37]. In summary, the improvements observed in pain levels and functional scores may be mirrored by chondral regeneration for at least up to a minimum of 3 years of follow-up, as assessed by MRI analyses.

The basic idea of MSC inducing cartilage regeneration was inspired by the approaches suggested by Pridie and Steadman [2,3], whose regenerative effects due to the differentiation of bone marrow MSC are well known [60,61,62]. Thus, autologous bone marrow MSC transplantation was proposed as a further evolution of this treatment approach. However, the current state of knowledge suggests that SVF has been found to have a higher content of MSC [63] with greater proliferative capacity [64] and more predictable cellular differentiation, while also being less invasive to harvest and carrying a lower risk of donor site morbidity including infection and pain [63,65]. Moreover, a recent meta-analysis also reported SVF to be more effective than bone marrow aspirate in pain reduction [66]. Recently, some research groups also investigated the effects of FDO combined with arthroscopic microfracture. Accordingly, three studies reported even better clinical outcomes when FDO was added to arthroscopic microfracture [60,61,62]. Leukocyte-poor PRP represents another substance pursuing a regenerative approach in OA treatment. Compared to FDO treatment, the methodological advantage of PRP is a lower material requirement and lower spatial demand for substrate harvesting and procession. Regarding the clinical outcomes, the current review involves three publications comparing FDO to PRP [22,34,50]. While two authors report similar functional improvements after 2 years [34,50], Khoury et al. report that cSVF outperformed PRP after 1 and 2 years in clinical and radiological parameters [22].

The strength of this systematic review is its focus on clinical mid-term results of FDO treatment in knee OA, while other reviews regarding FDO treatment discuss a wide range of indications [12,65,67], methodological details [9] cell compositions [68], or short-term results in knee OA [14]. This clear topic was chosen because the sustainability of symptom relief is deemed a major element in OA treatments. The main limitation of this review is that it contains plain descriptive outcomes of studies fulfilling the inclusion criteria regardless of their study design. Due to a lack of studies comparing FDO to a uniform control group, it was not possible to phrase a reasonable research question for a meta-analysis investigating the superiority of FDO. Therefore, there is a need for further RCT comparing FDO to commonly applied treatment options, i.e., PRP, to gain further insights into the efficacy of FDO. Regarding the included studies investigating FDO, the heterogeneity of study quality and treatment methods should be kept in mind when assessing the descriptive outcomes. Autologous fat tissue may be the common substrate of FDO, but it is ultimately applied after minimal or, to some extent, maximal manipulation [9]. The categories tSVF, cSVF and ASC aim to categorize the techniques, but even within these subcategories, a wide range of different methods are used.

Regarding tSVF, more than 17 different isolation systems are currently available and described in the medical literature [9,69]. This review shows large geographical differences in FDO methods: in Europe (and also in the United States), mainly tSVF is used because of very strict regulations if fat tissue is not obtained and applied during the same surgical procedure. However, emerging evidence suggests that mechanically disrupting adipose tissue (tSVF) has better regenerative effects compared to enzymatically processed lipoaspirate. Although the cell number and density are higher in the cSVF [70], the surface markers in tSVF are twice as high [71]. Moreover, in tSVF, adipocytes are selectively removed without damaging key components of the extracellular matrix. As mentioned before, an intact extracellular structure represents a niche and scaffold for cell modulation, migration, signaling, interaction, and differentiation [62,72,73]. While subcutaneous fat tissue is the basic material, further processing substantially defines its cell composition, biological properties, and terminology used for the respective method. In this regard, the chosen liposuction technique proves to have an impact on the cell composition and MSC quality [74,75,76]. Furthermore, the injection volume, the number and composition of cells or MSC content during application are not standardized. Moreover, individual patient prerequisites, e.g., age or stage of OA, also vary largely in the current literature. The patient’s mobilization, ranging between immediate full-weight bearing [32] and 2 months of non-weight bearing [35] in this review, might also represent an underestimated factor in providing the optimal environment for cartilage regeneration. Concomitant treatments or injections may also change or amplify the effect of FDO: PRP is often used to reduce inflammation and increase FDO’s paracrine effects (Supercharged Liparthroplasty), while fibrin glue should work as a degradable scaffold for ASC to differentiate into mature chondrocytes.

## 5. Conclusions

The present review demonstrates that the intra-articular administration of fat-derived orthobiologics (FDO) is a promising treatment option for patients with knee OA. It provides a wide range of beneficial mid-term results, including symptom reduction and preservation of the affected joint, which may postpone the need for arthroplasty. The review also revealed that 28 out of 29 studies showed pain reduction and functional improvement in knee OA treated with FDO. Moreover, a limited number of studies were able to demonstrate cartilage regeneration via MRI examinations and second-look arthroscopy. However, further research is necessary to determine the optimal processing, dosage and administration (including additional substances) of FDO as well as postoperative mobilization to define its ideal role in the treatment regimen of knee OA.

## Figures and Tables

**Table 1:** Search algorithm.

First Search Term	Boolean Operator	Second Search Term
“osteoarthritis” OR “arthritis”	AND	“adipose-derived stem cells” OR “fat grafting” OR “fat injection” OR “fat transfer” OR “fat transplantation” OR “mesenchymal stem cells” OR “mesenchymal stromal cells” OR “microfragmented adipose tissue” OR “stromal vascular fraction”

**Table 2:** Summary of the analyzed studies regarding the knee joint.

Authors	Study Design/LoE	Control Group	QoE Score	Patients/Joints	Age (y)	Sex (F/M)	OA Stage	FuP (y)	Fat Origin	Mech./Enzym.	Addit. Inject.	Inject./Focal Implantation	Arthorsc.	VAS Pre.	VAS Post.	PROMs, Funct. Param.		Pre	Post	Rad. FuP (*n*)	Second. Arthro.
Kim et al. [25]	retrosp./ IV	-	13/16	467/483	61 ± 6	333/150	I, II	7.2 ± 1.2	gluteal region	e (1d)	fibrin glue	focal	guidance	n/a	n/a	IKDC, Tenger	IKDC	39 ± 7	63 ± 9	-	-
Zhang et al. [31]	RCT/ I	HA	7/8	56/56	54 ± 14	42/14	II, III	5	abdomen	e	-	inject.	-	4.0 ± 1.5	2.9 ± 1.8	WOMAC	WOMAC	33 ± 22	27 ± 22	X-ray, MRI (51)	-
Kim et al. [32]	prosp./ III	-	16/16	11/11	61 ± 6	8/3	II, III	5	abdomen	e + c (3w)	-	inject.	-	≈6.6	≈2.6	WOMAC	WOMAC	≈60	≈35	X-ray, MRI (11)	-
Tantuway et al. [41]	RCT/ I	Saline	4/8	58/115	n/a	36/22	I, II, III	3	abdomen	m	-	inject.	-	8.4 ± 0.6	3.2 ± 0.9	KOOS	KOOS	43 ± 12	79 ± 6	-	-
Freitag et al. [36]	prosp./ II	-	12/16	27/27	54 ± 7	9/18	IV	3	abdomen	e + c (within 1w)	2. Inject. (6 m)	inject.	debrid., selective ME	5.6 ± 2.7	1.5 ± 1.4	KOOS, WOMAC, PGIC	KOOS-Pain	≈53	≈84	MRI (21)	-
Russo et al. [42]	retrosp./ IV	-	9/16	22/22	45 ± 11	8/14	II, III, IV	3	abdomen	m	-	inject.	-	≈5.8	≈2.8	KOOS, IKDC, Tegner	KOOS	≈52	≈80	-	-
Çimen et al. [43]	prosp./III	-	14/16	20/25	62 (50–76)	18/2	II, III	3	abdomen	m	-	inject.	-	6.7 ± 2.2	5.7 ± 2.2	WOMAC, Lysholm	WOMAC	63 ± 19	50 ± 26	-	-
Kim et al. [23]	retrosp./ III		15/24	37/39	58 ± 6	23/14	I, II	2.4 ± 0.3	gluteal region	e (1d)	-	focal	guidance	n/a	n/a	IKDC, Lysholm	IKDC	38 ± 8	62 ± 12	-	x
		→		17/17	58 ± 6	9/8	I, II	2.3 ± 0.3	gluteal region	e (1d)	fibrin glue	focal	guidance	n/a	n/a	IKDC, Lysholm	IKDC	36 ± 6	64 ± 12	-	
Kim et al. [24]	retrosp./ III		18/24	20/20	59 ± 3	13/7	I, II	2.4 ± 0.4	gluteal region	e (1d)	PRP	focal	guidance	n/a	n/a	IKDC, Tegner	IKDC	39 ± 9	56 ± 15	-	x
		→		37/68	59 ± 3	13/7	I, II	2.4 ± 0.3	gluteal region	e (1d)	fibrine glue + PRP	focal	guidance	n/a	n/a	IKDC, Tegner	IKDC	37 ± 5	65 ± 13	-	
Bakowski et al. [44]	retrosp./ IV	-	9/16	20/24	58 ± 8	21/16	I, II, III, IV	2.3 ± 0.5	abdomen	m	-	inject.	-	5.0 ± 2.2	4.1 ± 2.0	IKDC, WOMAC, KOOS, EQ-5D, 5× STS, TUG, 10 m WT	KOOS	59 ± 17	66 ± 16	-	-
Kim et al. [26]	prosp./ II	-	13/16	20/24	58 ± 6	9/11	I, II	2.3 (2–2.8)	gluteal region	e (1d)	fibrin glue	focal	guidance	n/a	n/a	IKDC, Tegner	IKDC	38 ± 7	67 ± 11	MRI (24)	-
Koh et al. [40]	retrosp./ IV	-	11/16	56/60	57 ± 5	34/22	I, II	2.2 ± 0.2	gluteal region	e (1d)	-	focal	debrid., guidance	n/a	n/a	IKDC, Lysholm	IKDC	38 ± 7	61 ± 11	-	x
Kim et al. [27]	retrosp./ IV	-	12/16	49/55	58 (48–69)	29/26	I, II	2.2 (2–3)	gluteal region	e (1d)	fibrin glue	focal	guidance	n/a	n/a	IKDC, Lysholm	IKDC	38 ± 6	67 ± 10	-	-
Ulivi et al. [33]	RCT/ I	arthrosc. debrid.	6/8	28/28	61 ± 8	n/a	III, IV	2.2 ± 0.8	abdomen or thigh	m	-	inject.	debrid.		−4	KOOS, WOMAC, SF-12	KOOS-Pain		≈+20	X-ray, mri (28)	-
Borg et al. [45]	retrosp./ IV	-	13/16	386/386	n/a	192/194	I, II, III, IV	2	abdomen or flank	m	-	inject.	-	n/a	n/a	OKS		†	†	-	-
Freitag et al. [46]	prosp./ II	-	14/16	297/297	59 ± 12	130/199	I, II, III, IV	2	abdomen or thigh	e + c (n/a)	-	inject.	-	5.2 ± 2.3	2.4 ± 2.2	WOMAC, KOOS, ROM	KOOS-Pain	≈58	≈78	-	-
Heidari et al. [47]	prosp./ II	-	13/16	220/344	n/a	95/125	III, IV	2	abdomen	m	-	inject.	-	n/a	n/a	OKS, EQ-5D		†	†	-	-
Screpis et al. [30]	prosp./ II	-	13/16	202/202	54 ± 9	105/97	I, II, III, IV	2.0 ± 0.8	abdomen or flank	m	-	inject.	-	≈5	≈6	KOOS	KOOS	≈55	≈75	-	-
Bistolfi et al. [48]	retrosp./ IV	-	12/16	78/78	60 ± 10	43/35	I, II, III	2	abdomen	m	-	inject.	-	7.1 ± 2.0	2.4 ± 2.9	IKS, Lysholm, FJS, KOOS	KOOS-Pain	41 ± 14	76 ± 15	-	-
Gobbi et al. [49]	retrosp./ IV	-	13/16	75/120	70	79/26	II, III, IV	2	abdomen or supragluteal region	m	-	focal and inject.	-	n/a	n/a	KOOS	KOOS-Pain	≈53	≈76	-	-
Fujita et al. [39]	retrosp./ IV	-	15/24	54/54	69 ± 10	40/14	II, III, IV	2	abdomen or gluteal region	e	-	inject.	-	6.5 ± 2.6	4.3 ± 2.4	WOMAC, ROM, Force	WOMAC	26 ± 12	17 ± 12	MRI (n/a)	-
Zaffagnini et al. [34]	RCT/ I	PRP	6/8	50/50	55 ± 12	25/28	I, II, III, IV	2	abdomen	m	-	inject.	-	6.6 ± 2.0	–1.5 ± 2.4	IKDC, KOOS, EQ-5D, EQ-VAS	KOOS-Pain	58 ± 16	10 ± 18	X-ray, MRI (50)	-
Gobbi et al. [50]	RCT/ I	PRP + HA (3×)	7/8	40/40	63 ± 13	23/17	0, I, II	2	abdomen	m	-	inject.	-	5.0 ± 2.0	4.0 ± 2.6	IKDC, Tegner, MKM, KOOS	KOOS-Pain	67 ± 18	73 ± 22	-	-
Koh et al. [28]	n/a	-	9/16	30/30	70 (65–80)	25/5	II, III, IV	2	gluteal region	e (1d)	PRP	focal	guidance	4.7 ± 1.6	1.7 ± 1.4	KOOS, Lysholm	KOOS-Pain	≈30	≈58	-	x
Yokota et al. [19]	prosp./ II		20/24	25/25	73 ± 9	20/5	II, III, IV	2	n/a	m	-	inject.	-	≈7.5	≈5.0	KOOS, OARSI	KOOS	≈38	≈54	X-ray (25)	-
		→		35/35	70 ± 9	28/7	II, III, IV	2	n/a	e + c (n/a)	-	inject.	-	≈7.2	≈3.8	KOOS, OARSI	KOOS	≈42	≈60	X-ray (35)	-
Khoury et al. [22]	retrosp./ IV	PRP	18/24	23/23	56 ± 9	7/16	I, II, III	2	n/a	e	-	inject.	-	6.0 ± 0.7	2.9 ± 0.7	KOOS	KOOS-Pain	50 ± 5	73 ± 4	MRI (23)	-
Koh et al. [29]	retrosp./IV	-	12/16	18/18	55 (41–69)	12/6	III, IV	2 (2–2.2)	infrapatellar fat pad	e	PRP	inject.	debr., selective ME, guidcance	4.8 ± 1.6	2.0 ± 1.1	WOMAC, Lysholm	WOMAC	50 ± 12	30 ± 9	MRI (18)	-
Jo et al. [35]	prosp./ III	-	14/16	17/17	62 ± 7	15/3	III, IV	2	abdomen	e + c (3w)	-	inject.	-	7.9 ± 0.2	4.6 ± 0.8	WOMAC, KOOS, IKS	KOOS-Pain	43 ± 4	76 ± 5	X-ray, MRI (17)	-
Boric et al. [37]	prosp./ III	-	12/16	10/10	69 ± 12	3/3	III, IV	2	abdomen	m	-	inject.	-	7.7 ± 1.4	3.4 ± 1.7	n/a	n/a	n/a	n/a	MRI (10)	-

Legends: Arthrosc. (arthroscopic technique); Addit. Inject (additional Injection); debrid. (debridement); EQ-5D (EuroQol 5 dimensions); F/M (female/male); FJS (Forgotten Joint Score); PROMs, Funct. Param. (Patient-Reported Outcome Measures, functional parameter/s); HA (hyaluronic acid); inject./focal implantation (intra-articular injection/focal implantation at arthritically degenerated cartilage); IKDC (Subjective International Knee Documentation Committee); IKS (International Knee Society score); Inject. (injection); K-L (Kellgren–Lawrence); KOOS (Knee Injury and Osteoarthritis Outcome Score); LoE (level of evidence); m/e/e + c (mechanical procession/enzymatical procession/enzymatical procession including cluture); Lysholm (Lysholm knee score); ME (meniscectomy); MKM (Marx knee measure); MQ (methodological quality); MRI (magnetic resonance imaging); n/a (not available); OA (osteoarthritis); OARSI (outcome measures in rheumatology–osteoarthritis research society international); OKS (Oxford knee score); PGIC (patients’ global impression of change scale); post. (postoperatively); pre. (preoperatively); prosp. (prospective study); PRP (platelet-rich plasma); RCT (randomized controlled trial); retrosp. (retrospective study); SF-12 (short form health survey-12); rad. FuP(n) (radiological follow-up (including number of patients)) ROM (range of motion); Second. arthro. (Secondary arthroscopy); Tegner (Tegner activity scale); TUG (timed up and go test); VAS (visual analogue scale for pain); WOMAC (western Ontario and McMaster universities); Y (years); 5× STS (5 times sit to stand test); 10 m WT (10 m walk test); → (indicating a second FDO method within one study); † (due to a gender-based assessment, no values for the total cohort are provided; however, improvements could be detected in both cohorts).

## Data Availability

Data is contained within the article.

## Abbreviations

ASC	Adipose-tissue-derived stromal/stem cells
cSVF	Cellular stromal vascular fraction
EQ-5D	EuroQol 5 dimensions
EQ-VAS	EuroQol 5 visual analogue scale
FDO	Fat-derived orthobiologics
FJS	Forgotten Joint Score
HA	Hyaluronic acid
ICRS	International Cartilage Repair Society Macroscopic Evaluation of Cartilage Repair
IKDC	Subjective International Knee Documentation Committee
IKS	International Knee Society score
K-L	Kellgren–Lawrence
KOOS	Knee Injury and Osteoarthritis Outcome Score
KKS	Knee Society score
LoE	Level of evidence
Lysholm	Lysholm knee score
ME	Meniscectomy
MRI	Magnetic resonance imaging
MKM	Marx knee measure
MOCART	MRI observation of cartilage repair tissue
MQ	Methodological quality
MSC	Mesenchymal stem cell
OA	Osteoarthritis
OARSI	Outcome measures in rheumatology–osteoarthritis research society international
OKS	Oxford knee score
PGIC	Oatients’ global impression of change scale
PRISMA	Preferred Reporting Items for Systematic Reviews and Meta-Analyses
PRP	Platelet-rich plasma
PROMs	Patient-Reported Outcome Measures
RCT	Randomized controlled trial
ROM	Range of motion
SF-12	Short form health survey-12
SVF	Stromal vascular fraction
Tegner	Tegner activity scale
tSVF	Tissue stromal vascular fraction
TUG	Timed up and go test
WOMAC	Western Ontario and McMaster universities
10 m WT	10 m walk test
5 × STS	5 times sit to stand test
